# Foods are differentially associated with subjective effect report questions of abuse liability

**DOI:** 10.1371/journal.pone.0184220

**Published:** 2017-08-31

**Authors:** Erica M. Schulte, Julia K. Smeal, Ashley N. Gearhardt

**Affiliations:** Department of Psychology, University of Michigan, Ann Arbor, Michigan, United States of America; University of Akron, UNITED STATES

## Abstract

**Objectives:**

The current study investigates which foods may be most implicated in addictive-like eating by examining how nutritionally diverse foods relate to loss of control consumption and various subjective effect reports. Subjective effect reports assess the abuse liabilities of substances and may similarly provide insight into which foods may be reinforcing in a manner that triggers an addictive-like response for some individuals.

**Design:**

Cross-sectional.

**Setting:**

Online community.

**Participants:**

507 participants (*n* = 501 used in analyses) recruited through Amazon MTurk.

**Measurements:**

Participants (*n* = 501) self-reported how likely they were to experience a loss of control over their consumption of 30 nutritionally diverse foods and rated each food on five subjective effect report questions that assess the abuse liability of substances (liking, pleasure, craving, averseness, intensity). Hierarchical cluster analytic techniques were used to examine how foods grouped together based on each question.

**Results:**

Highly processed foods, with added fats and/or refined carbohydrates, clustered together and were associated with greater loss of control, liking, pleasure, and craving. The clusters yielded from the subjective effect reports assessing liking, pleasure, and craving were most similar to clusters formed based on loss of control over consumption, whereas the clusters yielded from averseness and intensity did not meaningfully differentiate food items.

**Conclusion:**

The present work applies methodology used to assess the abuse liability of substances to understand whether foods may vary in their potential to be associated with addictive-like consumption. Highly processed foods (e.g., pizza, chocolate) appear to be most related to an indicator of addictive-like eating (loss of control) and several subjective effect reports (liking, pleasure, craving). Thus, these foods may be particularly reinforcing and capable of triggering an addictive-like response in some individuals. Future research is warranted to understand whether highly processed foods are related to these indicators of abuse liability at a similar magnitude as addictive substances.

## Introduction

Evidence is growing for the idea that some individuals may experience an addictive-like response to certain foods, which may contribute to obesity and eating-related problems [1–7]. The food addiction theory parallels the framework of substance-use disorders by positing that certain foods have an addictive potential and may interact with an individual’s proneness for addiction to result in an addictive-like phenotype [2, 8]. Consistent with substance-use disorders, indicators of food addiction include consumption in greater quantities than intended (i.e., loss of control over consumption), use despite negative consequences, persistent desire to cut down, and giving up important responsibilities or activities because of consumption [9, 10]. Individuals endorsing features of addictive-like eating exhibit similar characteristics as persons with substance-use disorders, such as difficulty regulating emotions, greater impulsivity, reward dysfunction, and altered dopamine signaling [11–14]. However, the food addiction construct is still controversial [15–17] and more research is needed.

One of the central components of the food addiction theory that warrants further empirical attention is the addictive potential of the food. This remains one of the greatest points of controversy associated with this topic [15, 18–20], in part due to the limited research conducted to date. In order for food addiction to be considered a substance-use disorder, then certain ingredients (e.g., sugar) or forms/quantities of ingredients (e.g., added sugar) need to be identified as having an addictive potential [15]. If foods are equally likely to be associated with addictive-like eating, an alternative hypothesis may be that food addiction is better conceptualized as a behavioral, eating addiction [21]. Yet, the current food addiction framework reflects a substance-use disorder perspective and theorizes that highly processed foods may have an addictive potential. In line with previous research investigating which foods are implicated in addictive-like eating [22, 23], the current study defined highly processed foods as having added amounts of fat and/or refined carbohydrates (e.g., cheeseburger, ice cream, cookies, French fries).

Presently, much of the evidence for which foods may be addictive comes from animal model studies. Rats appear to exhibit biobehavioral indicators of addiction in response to prolonged consumption of highly processed foods, (e.g., cheesecake, Oreo double stuf cookies), such as downregulation of dopamine receptors, binge consumption, elevated motivation to consume these foods despite negative consequences like foot shock, and cross-sensitization with amphetamine [24–28]. Importantly, rats do not display the same features in response to nutritionally balanced chow and will only binge-eat chow if primed with a taste of a highly processed food [25, 27, 29].

Consistent with animal research, the few studies in humans examining which foods are most associated with indicators of food addiction have observed that highly processed foods (e.g., pizza, chips, chocolate) were most reported as being consumed in an addictive manner [22, 30]. Notably, the most problematic highly processed foods seem to be high in both added fats and refined carbohydrates—a combination not found in minimally processed foods, defined in the current study as foods that do not contain added fats or refined carbohydrates. For instance, minimally processed foods may be naturally high in fat (e.g., nuts), carbohydrates (e.g., brown rice), or sugar (e.g., fruit), but only highly processed foods couple these characteristics together (e.g., ice cream). In this way, highly processed foods have an elevated quantity of rewarding ingredients (e.g., fat, refined carbohydrates) that are rapidly absorbed by the body (e.g., high glycemic load). [22]. Thus, highly processed foods may have an increased hedonic potential, relative to minimally processed foods.

Overall, animal models and preliminary findings in humans suggest that all foods are not equally associated with indicators of food addiction, and highly processed foods, which may have an elevated hedonic potential, appear to be most implicated in addictive-like eating [22, 30]. Yet, additional work is needed to elucidate whether highly processed foods relate to indexes of addiction potential in a similar manner as drugs of abuse. Thus, adapting approaches from the addiction literature to evaluate the possibly addictive nature of certain foods may be an appropriate next step in this line of research.

### Evaluating addictive potential

The addictive potential of a substance is assessed by indexes of abuse liability, or the likelihood that individuals with a propensity for addiction will compulsively consume the substance despite negative consequences [31, 32]. Substances with a high abuse liability (e.g., alcohol) are considered to be greatly rewarding and reinforcing, which perpetuates subsequent self-administration [31]. One established method for evaluating the reinforcing nature and abuse liability of substances is subjective effect reports, where individuals rate their experience consuming the substance on various facets of hedonic experience that may contribute to compulsive consumption [33–35]. Self-reported subjective experiences have been used to assess a substance’s abuse liability since the 1940s [36], and elevated abuse liability has been associated with greater self-administration of the substance in laboratory and naturalistic settings [37–40], suggesting that this measure exhibits predictive validity.

There are several facets of subjective experience that appear to be relevant across addictive substances. One of the most important indexes is euphoria, which assesses liking and pleasure derived from consuming the drug [35, 36, 41, 42]. Drugs with elevated euphoria are associated with greater abuse liability, as enjoyment during consumption may reinforce subsequent use [31, 32, 35]. Craving, conceptualized as a desire to use or consume more of the drug, also appears to be a central component of increased abuse liability [43], as increased craving for a substance may be indicative of greater motivation for future drug-seeking [44, 45]. Additionally, subjective effect reports frequently evaluate averseness of using the drug, which can reflect facets of oral consumption (e.g., taste) and acute physiological responses (e.g., dizziness, nausea). Another component often indexed is the intensity of the drug, which reflects the potency of the addictive agent with respect to intoxication effects or taste [43, 46]. Greater averseness and elevated intensity are both inversely related to abuse liability, as these negative experiences may reduce the reinforcing nature of the drug and the likelihood of repeated, compulsive use [43, 46].

### Approach

The current study will utilize cluster analytic techniques to explore how foods group together based on one characteristic of addictive-like consumption and several subjective effect report questions. Loss of control, defined as consumption/use in greater quantities or over longer periods of time than intended, is implicated in both addictive-like eating [2, 8] and substance use disorders [47]. Loss of control drug consumption is a commonly experienced symptom across substance-use disorders [48, 49], relates to motivation for drug-seeking behavior [50, 51], and increases relapse risk during efforts to cut down or abstain [50]. Similarly, loss of control is one frequently endorsed indicator of addictive-like eating [52], and loss of control over food consumption is related to severity of overeating episodes [53] and obesity [54], and future weight gain [55]. Given the higher prevalence of loss of control eating in previous community samples, relative to other symptoms of addictive-like eating (e.g., withdrawal), and the clinically relevant outcomes associated with loss of control consumption, the current study selected loss of control as one indicator of addictive-like eating behavior.

Food items will first be clustered based on the likelihood that they are reported to be associated with loss of control eating, in order to assess which foods are most related to this behavioral indicator of addictive-like use. Second, food items will be clustered based on five common subjective effect report questions: liking for taste, pleasure experienced during consumption, craving, averseness of taste, and intensity of taste. Lastly, the clusters formed by each subjective effect report question will be compared to the clusters yielded from reported loss of control eating to evaluate which indicators of abuse liability may be most informative for understanding one aspect of addictive-like food consumption.

The present work will add to the food addiction literature in two ways. First, this approach will contribute to understanding the variability within foods by examining factors that differentiate substances with a high abuse liability from those that have limited addictive potential. Subjective effect reports assessed in abuse liability approaches may be similarly useful for assessing whether foods vary in their reinforcing nature. If certain foods are more likely to be associated with indicators of abuse liability (e.g., greater craving and enjoyment, less averseness and intensity) than other foods, then this research may provide additional evidence for which foods may have a greater addictive potential. This study will also evaluate which subjective effect report questions may cluster food items in a similar way as a measure of loss of control over consumption. It may be that certain features of subjective experience (e.g., enjoyment) are more closely related to an indicator of addictive-like eating (loss of control) than others, which may provide insight into which subjective experiences may be most relevant to problematic eating behavior.

## Methods

### Ethics statement

The University of Michigan Health and Behavioral Sciences Institutional Review Board (IRB) approved the current study (HUM00118484) and determined it to be exempt from ongoing review. Given that the data was analyzed anonymously, the IRB determined that no written or oral consent form was required.

### Participants

Participants (*n* = 507) were recruited through Amazon Mechanical Turk (MTurk) to answer a survey about eating behavior. Participants were compensated 50 cents, a rate consistent with other MTurk studies [56]. Participants were excluded from analyses if they reported height and weight measures corresponding to a BMI outside of the feasible bounds (e.g., BMI < 10 or > 70) (n = 4) or for incorrectly answering “catch questions,” which have commonly-known answers (e.g. 2 + 2) designed to “catch” participants who respond without reading the questions carefully (n = 2). Of the 501 participants used in the analysis, 37.5% of participants identified as male (n = 188), 62.1% identified as female (n = 311), and 0.4% identified as other (n = 2). Participants varied in age from 18 to 74 years old (M = 34.5, SD = 10.8). Participant BMI was calculated using self-reported measures for height and weight, with values ranging from 14.77 (underweight) to 64.01 kg/m^2^ (obese), (M = 28.8, SD = 7.4; weight class distribution: underweight = 1.8%, normal weight = 29.9%, overweight = 33.9%, obese = 34.3%). Self-reported racial identification was as follows: 73.3% Caucasian/White (n = 367), 9.6% African American (n = 48), 6.6% Hispanic (n = 33), 6.0% Asian/Pacific Islander (30 participants), 1.4% American Indian (n = 7), 0.2% Arab (n = 1), and 3.0% identified as “other” (n = 15). The Yale Food Addiction Scale 2.0 was utilized to assess indicators of food addiction (see Gearhardt and colleagues [10] for scoring information). In the current sample, food addiction symptoms ranged from 0 to 11 (M = 2.39, *SD* = 3.12), and 14.6% of individuals met the diagnostic score threshold.

### Procedures

Through the online survey, participants were shown pictures of various food items, one at a time and in a randomized order, and asked to answer several questions about each food. They were instructed to respond to the questions while thinking about how they typically feel when they consume the pictured food.

#### Food stimulus set

Thirty nutritionally-diverse foods were included in the task, which were selected based on the one previous systematic study examining which foods are associated with addictive-like eating [22]. The foods in the present study varied slightly from Schulte and colleagues’ (2015) food stimulus set in two ways. First, five food items (fried chicken, rolls, crackers, strawberries, salmon) were removed to reduce participant burden, as their nutritional characteristics were already represented by other foods. Second, avocado was added to the food stimulus set to represent a high-fat fruit item, and donut replaced popcorn due to the variability in popular popcorn products (e.g., buttered popcorn versus light popcorn). The thirty foods in the current stimulus set varied across nutritional characteristics, such as calories, fat, carbohydrates, protein, sugar, and fiber. Fifteen foods were categorized as highly processed (e.g., muffin, pizza, cheeseburger), with added amounts of fats and/or refined carbohydrates, and fifteen foods were categorized as minimally processed (e.g., fruits, vegetables, meats), without added amounts of fats or refined carbohydrates. The nutrition facts were generated from registered dieticians affiliated with the University of Michigan’s Nutrition Obesity Research Center. Food pictures were selected from digitally available sources, were equal dimensions, and were all presented in color on a white background.

#### Subjective effect report questions

To further investigate which foods may be most associated with addictive-like eating, subjective effect questions were developed by the authors based on methodology utilized when evaluating the abuse liability of substances [36–38, 41–43, 46, 57]. For each pictured food item, participants reported on five facets of subjective experience: liking of the food’s taste, pleasure experienced during consumption, craving, averseness of the food’s taste, and intensity of the food’s taste. Additionally, participants rated how out of control they typically feel when consuming each food, as an indicator of problematic, addictive-like eating. The questions were presented as visual analog scales and in a randomized order for each food item. Table 1 details the wording and scaling for each question.

**Table 1:** Loss of control and subjective effect report questions.

How out of control do you feel when consuming this food? • 0 = Not out of control at all • 100 = Extremely out of control
How much do you like the taste of this food? • -100 = Dislike extremely • 100 = Like extremely
How much pleasure do you experience while consuming this food? • 0 = No pleasure • 100 = Extreme pleasure
How much do you typically crave this food? • 0 = No craving • 100 = Extreme craving
How aversive do you find the taste of this food? • 0 = Not at all aversive • 100 = Extremely aversive
How intense do you find the taste of this food? • 0 = Not at all intense • 100 = Extremely intense

### Data analytic plan

Cluster analytic techniques in SPSS Version 24.0 [58] were utilized in the current study to explore how food items group together based on facets of subjective experience. In order to account for the scaling variation on the visual analog scales, responses to the loss of control question and five subjective effect report questions were standardized to z-scores. Hierarchical cluster analyses using squared Euclidean distance and between-group linkage were conducted for each question. Clusters were formed by variables (e.g., craving rating for each food) rather than cases (e.g., craving rating from individual participant) in order to create clusters of the foods that generated similar responses on the subjective effect report questions, rather than clusters of individuals who reported similarly across all foods to each question.

For the cluster analyses performed on the loss of control question and each of the five subjective effect report questions, hierarchical analyses generated two- and three-cluster solutions. Across all questions, no three-cluster solutions appeared appropriate (e.g., less than three foods in the third cluster). For two-cluster solutions deemed as subjectively meaningful (e.g., greater than five foods in both clusters), independent-sample t-tests were conducted to evaluate if the mean rating on the subjective effect report question significantly differed between clusters 1 and 2. Statistically significant two-cluster solutions were retained. The research team then examined the clusters of each subjective effect report question to assess for any identical cluster solutions. It was observed that the clusters formed for two questions (liking of the food’s taste and pleasure during consumption) were identical. Given that these constructs are theoretically similar [59], both relating to enjoyment, a composite score was created by adding the unstandardized responses to these two questions. Then, the composite was standardized as a z-score and cluster analyses were again conducted. The resulting cluster analysis solution using the composite score yielded the same food clusters as the separate cluster analyses for the two individual questions and was thus retained.

Cluster solutions for each of the subjective effect report questions were examined individually, to elucidate how foods group together based on characteristics used to differentiate substances with a high versus low abuse liability. Additionally, the cluster solutions for each subjective effect report question were compared to the clusters formed based on loss of control eating, in order to investigate which facets of subjective experience (e.g., enjoyment) may most closely relate to an indicator of problematic, addictive-like eating behavior.

## Results

Table 2 summarizes the hierarchical clustering assignments of the 30 foods for loss of control and each subjective effect report question.

**Table 2:** Cluster assignments by question.

Food	Loss of Control	Liking + Pleasure	Craving	Averseness	Intensity	Highly Processed
Burger	1	1	1	1	1	Y
Cake	1	1	1	1	1	Y
Cereal	1	1	1	1	1	Y
Chips	1	1	1	1	1	Y
Chocolate	1	1	1	1	1	Y
Cookie	1	1	1	1	1	Y
Donut	1	1	1	1	1	Y
Fries	1	1	1	1	1	Y
Granola	1	2	2	1	1	Y
Gummy Candy	1	1	1	1	1	Y
Ice Cream	1	1	1	1	1	Y
Muffin	1	1	1	1	1	Y
Pizza	1	1	1	1	1	Y
Pretzels	1	1	1	1	1	Y
Soda	1	1	1	1	1	Y
Apple	2	2	2	1	1	N
Avocado	2	2	2	2	2	N
Bacon	1	1	1	1	1	N
Banana	2	2	2	1	1	N
Beans	2	2	2	2	2	N
Broccoli	2	2	2	2	1	N
Brown Rice	2	2	2	2	2	N
Carrots	2	2	2	1	1	N
Cheese	1	1	1	1	2	N
Chicken	2	1	1	1	1	N
Corn	2	2	2	1	1	N
Cucumber	2	2	2	2	2	N
Eggs	2	2	2	1	2	N
Nuts	1	2	2	1	1	N
Steak	1	1	1	1	1	N

### Loss of control

Clustering foods based on loss of control ratings produced two clusters that appear to separate the foods based on processing status. Cluster 1 contains the 15 highly processed foods included in the study, and four foods that are categorized as minimally processed, which are high in fat (bacon, cheese, nuts, and steak). Cluster 2 contains only foods categorized as minimally processed (e.g., fruits, vegetables, lean protein). An independent-sample t-test revealed a statistically significant difference in loss of control ratings between the two clusters (t = 10.06, p < 0.001, η2 = .68) (Cluster 1: M = 43.95, SD = 13.70; Cluster 2: M = 11.23, SD = 2.80), with Cluster 1 being associated with significantly greater reports of loss of control eating.

### Liking & pleasure

Clustering by the liking & pleasure composite score produced two clusters that seem to separate foods based on processing status. Cluster 1 contains 14 of the 15 processed foods, three minimally processed, high-fat foods (bacon, cheese, and steak), and one minimally processed and low-fat food (chicken). Cluster 2 contains all minimally processed foods except one highly processed food (granola). An independent-sample t-test revealed a statistically significant difference in liking/pleasure ratings between the two clusters (t = 5.04, p < 0.001, η2 = .48) (Cluster 1: M = 131.20, SD = 27.76; Cluster 2: M = 82.95, SD = 22.07), with significantly higher liking & pleasure composite scores being associated with Cluster 1.

### Craving

Clustering foods based on craving ratings resulted in two clusters that also appear to separate foods based on processing status, similar to the loss of control clustering. Cluster 1 contains the 15 highly processed foods included in the study, and the same four foods categorized as minimally processed that are high in fat observed in loss of control Cluster 1 (bacon, cheese, nuts, steak). Unlike loss of control, Cluster 1 for craving ratings also includes chicken, which is minimally processed. Akin to loss of control, Cluster 2 for craving ratings also contains only minimally processed foods. An independent-sample t-test again revealed a statistically significant difference in craving ratings between the two clusters (t = 4.90, p < 0.001, η2 = .46) (Cluster 1: M = 53.62, SD = 12.05; Cluster 2: M = 34.54, SD = 7.28), with Cluster 1 exhibiting significantly higher reports of craving.

### Averseness

Clustering by averseness of food taste produced two clusters, varying in size. Cluster 1 contains 25 of the 30 foods in the study, including all 15 highly processed foods. Cluster 2 contains only five minimally processed foods (avocado, beans, broccoli, cucumber, and brown rice). An independent-sample t-test revealed a statistically significant difference in averseness ratings between the two clusters (t = -4.76, p < 0.001, η2 = .45) (Cluster 1: M = 19.23, SD = 3.47; Cluster 2: M = 27.03, SD = 2.44), with Cluster 2 being associated with higher ratings of averseness.

### Intensity

Clustering by intensity of food taste produced two clusters that appear similar to those yielded by clustering by averseness. Cluster 1 contains 24 of the 30 foods included in the study, including all 15 highly processed foods. Cluster 2 contains six minimally processed foods (cheese, eggs, avocado, beans, cucumber, brown rice). An independent-sample t-test revealed a statistically significant difference in intensity ratings between the two clusters (t = 2.36, p = 0.03, η2 = .17) (Cluster 1: M = 55.19, SD = 12.75; Cluster 2: M = 41.29, SD = 13.59), with Cluster 1 demonstrating significantly higher intensity responses.

## Discussion

The current study clustered nutritionally diverse foods based on loss of control, a feature of addictive-like consumption, and five subjective effect report questions that have been used to investigate the abuse liability of substances [36–38, 41–43, 46, 57]. This approach was used to examine how foods vary in their associations with indicators of abuse liability (e.g., enjoyment, craving) to elucidate which foods may have the greatest potential to engage addictive-like processes. Further, the clusters formed by each subjective effect report question were compared to the clusters yielded based on reported loss of control in order to evaluate which facets of abuse liability may be most informative to understanding which foods are most implicated in addictive-like eating.

### Loss of control

Loss of control over consumption is a feature of substance-use disorders and addictive-like eating [2, 47, 60] associated with clinically relevant outcomes of drug use and overeating [50, 53, 55]. Clusters produced for loss of control suggested that highly processed foods (e.g., chocolate, fries) are associated with greater reported loss of control eating, as all 15 highly processed foods clustered together. This finding supports previous research that observed highly processed foods were most associated with all behavioral indicators of addictive-like eating [22, 30] and provides further evidence that highly processed foods may be most likely to trigger addictive-like responses in susceptible individuals.

In addition, four foods categorized as minimally processed clustered with the 15 highly processed foods as being related to greater loss of control consumption. These foods (bacon, cheese, nuts, steak) are all naturally high in fat and frequently contain added amounts of salt, which some research has suggested may increase the hedonic appeal of food [61–63]. However, the loss of control ratings for these four minimally processed foods descriptively appear to be, on average, lower relative to highly processed foods but higher than minimally processed foods naturally lower in fat (e.g., fruits, vegetables, chicken breast) (see Table A in S1 File for mean loss of control ratings for each food). Thus, these clusters may suggest that foods fall on a spectrum of risk to be associated with loss of control over consumption, a feature of addictive-like eating. Consistent with previous research, highly processed foods (e.g., chips, pizza) appear to be most problematic, as these foods were most related to reported loss of control over consumption, whereas minimally processed foods naturally low in fat (e.g., fruits, vegetables, lean protein) seem to be least related. Further, within the cluster of foods reported to be most associated with loss of control, highly processed foods were on average reported to be more problematic than minimally processed foods high in fat.

### Enjoyment and craving

Two mechanisms that are closely related to abuse liability of substances are enjoyment and craving, with higher reports being related to greater addictive potential [31, 32, 41, 42, 46]. Clusters yielded from two indexes of enjoyment (liking, pleasure) paralleled groupings from the loss of control question, with few exceptions. Nuts and granola were both associated with high loss of control but lower enjoyment, whereas chicken was reported as being highly enjoyed, despite being minimally linked to loss of control eating (see Table B in S1 File for mean enjoyment ratings of each food). Clustering foods based on craving yielded nearly identical clusters as loss of control, with all 15 highly processed foods and four high-fat, minimally processed foods (cheese, bacon, nuts, steak) associated with greater craving reports (see Table C in S1 File for mean craving ratings of each food). The one difference between craving and loss of control clusters was that chicken was reported as marginally related to loss of control but highly craved.

Overall, most foods associated with loss of control eating are also highly craved and enjoyed, suggesting that enjoyment and craving are important subjective experiences associated with problematic food consumption. This parallels studies assessing the abuse liability of addictive substances, observing that greater liking, pleasure, and craving are related to increased likelihood of compulsive drug use [31, 32, 35, 43]. Enjoyment seems to enhance the reinforcing nature of the substance, whereas craving may index motivation for continued drug-seeking behavior [44]. Similarly, greater liking and craving appear to contribute to the hedonic and motivational properties of a food [59, 64]. Thus, the current study provides support that akin to findings in addictive disorders, enjoyment and craving may be informative for understanding which foods may be most reinforcing and likely to be associated with loss of control eating, a feature of addictive-like consumption. This work also offers further evidence that highly processed foods are most closely related to these indicators of abuse liability and minimally processed foods low in fat demonstrating little association. Additionally, minimally processed foods that may contain added salt (e.g., cheese, bacon) clustered with highly processed foods, though their mean ratings were comparatively lower on average, suggesting a moderate association.

The one inconsistent finding was that chicken was reported as being highly craved and enjoyed. Chicken is a staple in many Americans’ diets and is trending to usurp beef as the most commonly consumed protein in the United States [65, 66], and thus, it seems logical that such a prevalent food would be rewarding. There is also significant variability in how chicken may be prepared, which may contribute to elevated craving and enjoyment scores. For instance, chicken is often paired with added fats and/or refined carbohydrates (e.g., chicken sandwich on white bread with mayonnaise), which would may increase its likelihood to engage reward-related processes. Given that chicken was not in the cluster of the foods highly associated with loss of control, it may be that this commonly eaten food is craved and enjoyed but unlikely to trigger this feature of addictive-like eating. This finding raises an empirical question to investigate possible factors that protect some foods (e.g., chicken), but not others (e.g., pizza, chocolate), that are enjoyed and craved from being implicated in loss of control eating.

### Averseness and intensity

Cluster analyses of subjective effect reports assessing averseness of the food’s taste and intensity of the food’s taste were less informative of which foods were related to greater loss of control consumption. The clusters formed for each of these two questions consisted of uneven groups, as few foods were rated as highly averse or low in intensity (see Table D in S1 File (averseness) and Table E in S1 File (intensity) for mean ratings of each food). Further, the effect size for the difference between the two intensity clusters was small. With respect to the abuse liability of substance-use disorders, questions of averseness and intensity can be directed at the experience (e.g., physiological symptoms like lightheadedness) and consequences of consuming the drug (e.g., intoxication) [36, 67]. For substances consumed orally (e.g., oral tobacco), the averseness and intensity of the taste are considered to be particularly important for abuse liability [46]. Given that foods do not induce intoxication or acute physiological responses akin to drugs of abuse, the current study assessed only averseness and intensity of taste.

While the specific subjective effect report questions evaluating averseness and intensity may vary across substances to reflect relevant symptoms (e.g., inclusion of questions assessing taste intensity for oral tobacco), averseness and intensity are typically negatively related to abuse liability [43, 46]. However, with respect to food, it may be that averseness and intensity of foods’ tastes reflect taste preferences, where only few foods were commonly reported as highly averse (e.g., beans) or low in taste intensity (e.g., brown rice). Further, palatability is a central feature that promotes daily food consumption broadly, with minimally processed foods needing to be palatable from an evolutionary perspective and the palatability of highly processed foods promoting industry sales. In contrast, for addictive substances consumed orally (e.g., alcohol, oral tobacco), recreational use is likely promoted as long as the substance is not averse.

Yet, while few food items were reported to have a highly averse taste, a majority of foods were clustered into a group with elevated taste intensity. With respect to food, a greater taste intensity (e.g., intense sweet taste) may be palatable, whereas greater taste intensity of orally consumed substances (e.g., oral tobacco) may be averse. However, there may also be facets of averseness and intensity that are particularly relevant to problematic food consumption, such as negative physical (e.g., sluggishness, stomach discomfort) and emotional (e.g., disgust) consequences of addictive-like eating. Thus, the current findings suggest that subjective effect reports assessing averseness and intensity of foods’ tastes do not seem to meaningfully separate which foods are implicated in loss of control consumption, though future research may consider assessing additional features of averseness and intensity that may be especially applicable to addictive-like eating (e.g., bloating, disgust).

### Future directions and limitations

The current study provides evidence using self-report methods that foods vary in their association with one indicator of addictive-like eating, loss of control, and subjective experiences implicated in the abuse liability of drugs. This provides further support that highly processed foods may be more reinforcing and may translate to these foods being more likely to be consumed problematically than minimally processed foods. However, these findings do not conclude that highly processed foods are addictive, as no comparison can be drawn for the magnitude of the subjective experiences to foods in the present work relative to responses towards addictive substances. Future research may consider assessing subjective effect reports towards highly processed foods and an addictive substance among individuals who experience problems related to both food and drug use (e.g., individuals with addictive-like eating behavior who also exhibit alcohol-use disorder). This study design would be appropriate for comparing the abuse liability of highly processed foods relative to drugs of abuse. If highly processed foods are reported to be similarly reinforcing as addictive substances on indexes of subjective experience, then it may be appropriate to conclude that highly processed foods have an abuse liability for some individuals. Another important direction for future research may be to explore whether the subjective experience of foods vary based on food addiction symptomology, as the current sample was underpowered to conduct cluster analyses with the 73 individuals who met for food addiction based on the YFAS 2.0. Recruitment of a larger sample of persons with addictive-like eating may provide greater insight into which foods are most associated with facets of abuse liability.

The present study had several limitations, such as the use of self-report data and recruitment techniques that may have targeted individuals with an interest in answering questions about eating behavior. The generalizability of the current sample may be limited from the use of MTurk, which appears to recruit a diverse, but not nationally representative sample [56], and this emphasizes the need to replicate the current findings through further work. Further, the current sample was an online community sample, and replication is needed in relevant clinical populations (e.g., individuals with food addiction). While the present approach allowed for online data collection from a large sample size, it was not possible to have participants provide subjective effect ratings while consuming each food. Preston and Walsh [57] outline gold-standard procedures for evaluating abuse liability using subjective effect reports, which includes having individuals consume a standardized portion of a drug prior to providing self-report subjective effect ratings. Integration of behavioral and self-report methods of abuse liability represents an immediate next step in furthering the understanding of how foods vary in their association with indicators of addictive potential.

Another limitation of the present work is that not all features of subjective experience were assessed. In order to prioritize including a wide range of nutritionally diverse foods without over burdening participants, we selected five subjective effect report questions that index common measures of abuse liability. However, subjective experience questionnaires often consist of numerous items for each construct (e.g., liking for taste, liking for mouth feel) and additional facets of abuse liability (e.g., behavioral economics) [37]. Thus, future studies may aim to reduce the food items to include only those that clustered highly with indicators of problematic eating and abuse liability in the current study but include a wider range of subjective effect report questions. Similarly, the current approach included one common facet of addictive-like eating, loss of control, which may limit generalizability to all indicators of food addiction (e.g., use despite negative consequences, tolerance). One extension of the current work may be to explore how the subjective experience of consuming various foods relates the Yale Food Addiction Scale [9, 10], which is a validated measure of addictive-like eating. Further, while the foods included in the current work were diverse across nutritional characteristics (e.g., calories, fat, carbohydrates, protein) and taste profiles (e.g., sweet, salty), they are not exhaustive. Thus, future work may consider expanding the number of foods surveyed in order to more comprehensively capture individual preferences.

### Summary

The present work adapted methodology from the addiction literature to evaluate which foods are most associated with indicators of abuse liability (e.g., craving) and one feature of addictive-like eating behavior (loss of control). Collectively, the findings support that foods may fall on a risk spectrum of how likely they are to be implicated in problematic, addictive-like eating. Highly processed foods appear to be the most implicated in loss of control eating, supporting previous research that these foods may be most implicated in addictive-like eating behavior. Further, highly processed foods were associated with greater reported craving and enjoyment, suggesting that these foods may be most reinforcing and have the greatest potential for abuse liability. In contrast, the majority of minimally processed foods (e.g., fruits, vegetables) were least related. However, several minimally processed foods that may contain added sodium (e.g., cheese, bacon) clustered with highly processed foods. On average, these foods were rated below highly processed foods, suggesting a moderate association with reported loss of control eating and subjective effect reports. As has been suggested by previous researchers [61–63], added salt may elevate the reinforcing nature of food, akin to added fat and refined carbohydrates. Future studies may consider categorizing foods as highly processed if they contain added fats, refined carbohydrates, and/or salt and determine whether certain quantities/combinations of these ingredients or related characteristics (e.g., glycemic load) are particularly associated with the possible addictive potential of highly processed foods. Notably, foods varied in their associations with facets of abuse liability, providing further support for a substance-use disorder conceptualization of food addiction, where foods high in fat, refined carbohydrates, and/or salt may trigger an addictive response in certain individuals. If addictive-like eating were more appropriately conceptualized as a behavioral addiction, as has been suggested by other researchers [21], it may have been expected that all foods related similarly to features of addictive potential. Lastly, craving and enjoyment clustered foods most similarly as loss of control, an indicator of addictive-like eating, whereas intensity and averseness of the foods’ taste were less informative. Thus, further work examining the potential abuse liability of food may focus on the aspects of subjective experience most relevant to addictive-like food consumption and explore additional subjective effect responses that may be particularly relevant to overeating (e.g., averseness of physical consequences like stomach discomfort).

## Supporting information

S1 File **Table A. Mean Loss of Control Ratings by Food.** Ratings can range from 0 to 100. **Table B. Mean Enjoyment (Liking + Pleasure) Ratings by Food.** Ratings can range from -100 to 200. **Table C. Mean Craving Ratings by Food.** Ratings can range from 0 to 100. **Table D. Mean Averseness Ratings by Food.** Ratings can range from 0 to 100. **Table E. Mean Intensity Ratings by Food.** Ratings can range from 0 to 100.
(DOCX)
