# Supplementary material for: Foods are differentially associated with subjective effect report questions of abuse liability
Source: PLoS One. 2017 Aug 31;12(8):e0184220. doi: 10.1371/journal.pone.0184220 (PMC5578654; doi:10.1371/journal.pone.0184220)
Supplement: S1 File — Table A. Mean Loss of Control Ratings by Food. Ratings can range from 0 to 100. Table B. Mean Enjoyment (Liking + Pleasure) Ratings by Food. Ratings can range from -100 to 200. Table C. Mean Craving Ratings by Food. Ratings can range from 0 to 100. Table D. Mean Averseness Ratings by Food. Ratings can range from 0 to 100. Table E. Mean Intensity Ratings by Food. Ratings can range from 0 to 100. (DOCX) [file pone.0184220.s001.docx]

**Table A. Mean Loss of Control Ratings by Food.**

| Food | Mean | Processed |
| --- | --- | --- |
| Ice Cream | 58.65 | Y |
| Cookie | 58.54 | Y |
| Chocolate | 57.70 | Y |
| Fries | 57.33 | Y |
| Cake | 57.10 | Y |
| Donut | 56.60 | Y |
| Pizza | 56.31 | Y |
| Chips | 55.96 | Y |
| Burger | 51.16 | Y |
| Soda | 44.82 | Y |
| Bacon | 40.89 | N |
| Gummy Candy | 39.70 | Y |
| Cheese | 34.67 | N |
| Muffin | 34.26 | Y |
| Cereal | 33.34 | Y |
| Steak | 29.87 | N |
| Nuts | 25.39 | N |
| Pretzels | 24.47 | Y |
| Granola | 18.34 | Y |
| Chicken | 16.00 | N |
| Eggs | 15.14 | N |
| Avocado | 12.62 | N |
| Corn | 12.26 | N |
| Banana | 12.16 | N |
| Brown Rice | 10.83 | N |
| Apple | 10.70 | N |
| Beans | 10.26 | N |
| Carrots | 8.78 | N |
| Broccoli | 7.86 | N |
| Cucumber | 6.99 | N |

Ratings can range from 0 to 100.

**Table B. Mean Enjoyment (Liking + Pleasure) Ratings by Food.**

| Food | Mean | Processed |
| --- | --- | --- |
| Pizza | 162.45 | Y |
| Ice Cream | 160.50 | Y |
| Chocolate | 159.31 | Y |
| Cookie | 157.05 | Y |
| Fries | 149.95 | Y |
| Cheese | 147.68 | N |
| Cake | 145.55 | Y |
| Donut | 143.72 | Y |
| Burger | 142.08 | Y |
| Bacon | 138.27 | N |
| Steak | 137.58 | N |
| Chips | 127.37 | Y |
| Chicken | 120.94 | N |
| Muffin | 118.63 | Y |
| Apple | 117.27 | N |
| Eggs | 107.41 | N |
| Banana | 106.75 | N |
| Cereal | 105.39 | Y |
| Soda | 95.69 | Y |
| Granola | 88.99 | Y |
| Nuts | 88.29 | N |
| Corn | 87.56 | N |
| Broccoli | 82.35 | N |
| Avocado | 78.96 | N |
| Gummy Candy | 77.51 | Y |
| Cucumber | 74.92 | N |
| Pretzels | 71.86 | Y |
| Carrots | 69.61 | N |
| Beans | 50.51 | N |
| Brown Rice | 42.76 | N |

Ratings can range from -100 to 200.

**Table C. Mean Craving Ratings by Food.**

| Food | Mean | Processed |
| --- | --- | --- |
| Pizza | 70.45 | Y |
| Chocolate | 69.96 | Y |
| Ice Cream | 66.25 | Y |
| Cookie | 63.21 | Y |
| Cheese | 62.99 | N |
| Fries | 62.88 | Y |
| Burger | 59.71 | Y |
| Steak | 57.25 | N |
| Cake | 54.91 | Y |
| Bacon | 53.57 | N |
| Chicken | 52.36 | N |
| Chips | 52.33 | Y |
| Donut | 49.54 | Y |
| Soda | 45.00 | Y |
| Banana | 44.85 | N |
| Cereal | 44.80 | Y |
| Eggs | 44.11 | N |
| Apple | 43.78 | N |
| Muffin | 37.96 | Y |
| Nuts | 37.60 | N |
| Avocado | 36.00 | N |
| Corn | 34.97 | N |
| Broccoli | 34.33 | N |
| Granola | 33.29 | Y |
| Gummy Candy | 32.24 | Y |
| Pretzels | 29.77 | Y |
| Carrots | 28.82 | N |
| Cucumber | 28.12 | N |
| Beans | 25.27 | N |
| Brown Rice | 23.28 | N |

Ratings can range from 0 to 100.

**Table D. Mean Averseness Ratings by Food.**

| Food | Mean | Processed |
| --- | --- | --- |
| Beans | 30.81 | N |
| Soda | 30.30 | Y |
| Avocado | 27.95 | N |
| Brown Rice | 26.34 | N |
| Broccoli | 25.36 | N |
| Cucumber | 24.71 | N |
| Gummy Candy | 24.46 | Y |
| Nuts | 21.73 | N |
| Cereal | 21.23 | Y |
| Carrots | 21.11 | N |
| Bacon | 21.03 | N |
| Banana | 21.01 | N |
| Granola | 20.74 | Y |
| Eggs | 20.74 | N |
| Pretzels | 20.42 | Y |
| Chips | 20.38 | Y |
| Burger | 20.14 | Y |
| Steak | 18.60 | N |
| Muffin | 18.42 | Y |
| Donut | 18.22 | Y |
| Cheese | 18.17 | N |
| Chicken | 17.93 | N |
| Fries | 17.16 | Y |
| Cake | 17.12 | Y |
| Corn | 16.96 | N |
| Pizza | 16.28 | Y |
| Chocolate | 15.08 | Y |
| Ice Cream | 15.02 | Y |
| Cookie | 14.65 | Y |
| Apple | 13.95 | N |

Ratings can range from 0 to 100.

**Table E. Mean Intensity Ratings by Food.**

| Food | Mean | Processed |
| --- | --- | --- |
| Chocolate | 74.04 | Y |
| Bacon | 71.31 | N |
| Cake | 70.30 | Y |
| Pizza | 70.25 | Y |
| Ice Cream | 69.23 | Y |
| Steak | 68.34 | N |
| Burger | 67.12 | Y |
| Cookie | 65.44 | Y |
| Cheese | 63.85 | N |
| Donut | 63.12 | Y |
| Soda | 62.84 | Y |
| Fries | 59.78 | Y |
| Chips | 57.23 | Y |
| Muffin | 49.85 | Y |
| Banana | 49.53 | N |
| Apple | 48.93 | N |
| Cereal | 48.12 | Y |
| Chicken | 47.70 | N |
| Gummy Candy | 47.25 | Y |
| Avocado | 46.46 | N |
| Broccoli | 44.43 | N |
| Eggs | 43.78 | N |
| Granola | 41.15 | Y |
| Nuts | 41.10 | N |
| Carrots | 36.68 | N |
| Beans | 35.84 | N |
| Corn | 35.50 | N |
| Pretzels | 35.34 | Y |
| Cucumber | 33.65 | N |
| Brown Rice | 24.17 | N |

Ratings can range from 0 to 100.
